# Acupuncture therapy for isolated ischemic oculomotor nerve palsy: A case report

**DOI:** 10.1097/MD.0000000000037850

**Published:** 2024-04-19

**Authors:** Guan Xu, Kelin He, Xiao Ye, Songhao Ning, Quanai Zhang

**Affiliations:** a Zhejiang Chinese Medical University, Hangzhou, China; b Department of Acupuncture and Moxibustion, The Third Affiliated Hospital of Zhejiang Chinese Medical University, Hangzhou, China; c Confucius Institute, University of Coimbra, Coimbra, Portugal.

**Keywords:** acupuncture therapy, case report, isolated ischemic oculomotor nerve palsy, microvascular ischemia

## Abstract

**Background:**

Isolated ischemic oculomotor nerve palsy as a type of ophthalmic disease is rarely observed in clinical practice. Quality of life is frequently impacted by isolated ischemic oculomotor nerve palsy due to its lack of treatment options and long-term visual impairment. We describe an acupuncture-treated instance of isolated ischemic oculomotor paralysis.

**Methods:**

Acupoints including Jingming (BL 1), Chengqi (ST 1), Cuanzhu (BL 2), and Sizhukong (TE 23) on the right side, and bilateral Fengchi (GB 20), Waiguan (TE 5), Hegu (LI 4), and Zulinqi (GB 41) were selected for needling. Each treatment lasted for 30 minutes, once every other day. Acupuncture treatment was administered for a total of 11 times.

**Results:**

Acupuncture is a promising treatment option for isolated ischemic oculomotor nerve palsy.

**Conclusions:**

Ischemic oculomotor nerve paralysis can affect the quality of life of patients. Acupuncture intervention can promote the recovery of the disease is a very effective treatment measure.

## 1. Introduction

Isolated ischemic oculomotor nerve palsy is caused by ischemia of the nutrient vessels of the oculomotor nerve.^[1]^ Predominantly, it manifests as ptosis as well as limited upward, downward, and inward eye movement, but also as double vision, with normal pupil size and the light reflex.^[1]^ Microvascular ischemia is the primary cause of oculomotor nerve palsy, accounting for approximately 42% of all cases.^[2]^ This condition is predominantly observed in elderly patients.^[3]^ Clinical observation is the first-line treatment of isolated ischemic oculomotor nerve palsy, based on the assumption that it may spontaneously elapse within 8–20 weeks.^[1,4]^ Importantly, to reduce the risk of arteriosclerosis, the patients’ conditions should be monitored by their internists during the observation period.^[1]^ However, the condition may progress during the prolonged clinical observation, impacting the patient’s quality of life. Hence, effective therapeutic measures are urgently needed to expedite the recovery. Pertinent research indicates that acupuncture, with its simplicity and negligible adverse effects, is fairly effective in treating oculomotor nerve palsy.^[5]^ We report a case of a patient with isolated ischemic oculomotor nerve palsy, who did not show any noticeable improvement in clinical symptoms after 2 weeks of pharmaceutical intervention. After undergoing consistent acupuncture treatment for 3 weeks, the patient recovered significantly faster, and his quality of life greatly improved. Therefore, we carried out a retrospective analysis of this patient’s case.

## 2. Case report

### 2.1. Case presentation

**Figure 1.**
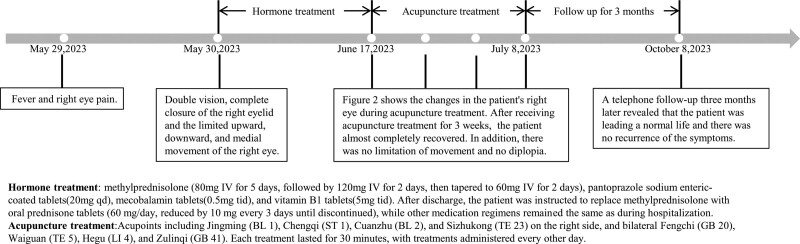
Timeline of the clinical manifestations and the therapeutic interventions of the present case.

On June 17, 2023, a 62-year-old male patient presented with a history of right palpebral ptosis persisting for over half a month, accompanied by double vision. The patient had experienced fever and right eye pain half a month earlier. The following morning, he noticed double vision and slight ptosis on the right side. By the afternoon of the same day, the degree of the palpebral ptosis had progressively increased. As a result, a complete closure of the right eyelid ensued, as well as the limited upward, downward, and medial movement of the right eye. Eventually, the patient was admitted to a local hospital. Routine blood tests, coagulation studies, glycated hemoglobin, toluidine red unheated serum test + treponema pallidum gelatin particle agglutination test (serum), hepatitis markers, antineutrophil cytoplasmic antibodies, antinuclear antibody, complete vitamin profile, cerebrospinal fluid biochemistry, and cerebrospinal fluid bacterial smear tests all yielded normal results. Orbital enhanced magnetic resonance imaging ruled out the possibility of a tumor. A cranial CT report indicated the presence of arteriosclerosis. Based on clinical manifestations and auxiliary examinations, the patient was diagnosed with oculomotor nerve palsy. During hospitalization, the patient was administered methylprednisolone (80 mg IV for 5 days, followed by 120 mg IV for 2 days, then tapered to 60 mg IV for 2 days), pantoprazole sodium enteric-coated tablets, mecobalamin tablets, and vitamin B1 tablets. After discharge, the patient was instructed to replace methylprednisolone with oral prednisone tablets (60 mg/day, reduced by 10 mg every 3 days until discontinued), while other medication regimens remained the same as during hospitalization. The patient, with a 10-year history of hypertension, currently under treatment with oral amlodipine tablets (5 mg/day), presented with a blood pressure of 146/78 mm Hg. The patient has no history of trauma, diabetes, hepatitis, tuberculosis, or hereditary diseases in the family. The clinical manifestations and treatment intervention schedule of this case are shown in Figure 1.

### 2.2. Investigations

The physical examination revealed that the patient’s right upper eyelid was completely closed, with a palpebral fissure width of 0 mm. The patient was unable to raise it voluntarily. The eye exhibited limited vertical movement of approximately ±10° and limited medial movement of about 10°, while lateral movement was not restricted. The patient experienced vertical diplopia (with the phantom image above the true image). The pupils were equal, round, and approximately 3 mm in diameter on both sides, with a brisk pupillary light reflex. No exo- nor enophthalmos were observed, and no visual field defects, nor nystagmus were detected. Color vision and accommodative reflexes were normal in both eyes, with a weaker convergence reflex in the right eye compared to the left. The neostigmine test was negative. Sensation in both the deep and superficial areas of the face was symmetrical. The neck was supple without resistance. Limb strength and muscle tone were normal, and pathological signs were negative bilaterally.

### 2.3. Treatment

Given that prednisone tablets are ineffective for oculomotor nerve palsy induced by microvascular ischemia and carry the risk of affecting metabolic regulation, the patient was advised to discontinue prednisone before acupuncture treatment while continuing the intake of other medications.^[6]^ The patient agreed to this treatment plan. The acupuncture treatment was administered by a professional acupuncturist. Acupoints were located as per the *Science of Acupuncture and Moxibustion*,^[7]^ including Jingming (BL 1), Chengqi (ST 1), Cuanzhu (BL 2), and Sizhukong (TE 23) on the right side, and bilateral Fengchi (GB 20), Waiguan (TE 5), Hegu (LI 4), and Zulinqi (GB 41). After local disinfection of the acupoints, single-use acupuncture needles of 0.25 × 25 mm were used for the acupuncture treatment. Each treatment lasted for 30 minutes, once every other day. The location of acupoints and operations are listed in Table 1.

**Table 1:** Acupuncture points, locations, and specific operations.

Acupuncture points	Location	Method of insertion
Jingming (BL 1)	In the depression superior to the inner canthus	Have a patient close his eyes. With left hand gently push the eyeball toward the lateral side, with right hand slowly insert needle perpendicularly 0.5–1.0 cun along the orbital wall. It is not advisable to rotate or lift and thrust the needle (or only rotate or lift and thrust slightly). To avoid bleeding, press the punctures site momentarily after withdrawing the needle
Chengqi (ST 1)	With the eyes looking straight forward, the point is directly below the pupil of the eye, between the eyeball and the infraorbital ridge	Push the eyeball upward slightly with left thumb and puncture perpendicularly and slowly 0.5–1.5 cun along the infraorbital ridge. It is not advisable to manipulate the needle with large amplitude, to avoid injuring the blood vessel resulting in hematoma. Press the point for 1 min to avoid bleeding when withdrawing the needle
Cuanzhu (BL 2)	In the depression on the medial end of eyebrow, on the supraorbital notch	Subcutaneous or oblique insertion 0.5–0.8 cun
Sizukong (TE 23)	In the depression at the lateral end of the eyebrow	Subcutaneous insertion 0.3–0.5 cun
Fengchi (GB 20)	On the nape, below the occiput, at the level of Fengfu (GV 16), in the depression between the upper portion of m. sternocleidomastoideus and m. trapezius	Oblique insertion 0.8–1.2 cun toward the tip of the nose with the tip of the needle slightly downward, or subcutaneous insertion through Fengfu (GV 16). There is the medulla oblongata toward the middle in the deeper layer; the angle and depth of the needle must be strictly controlled
Waiguan (TE 5)	On the dorsal aspect of the forearm, on the line connecting Yangchi (TE 4) and tip of the elbow, 2 cun above the transverse crease of the wrist between the ulna and radius	Perpendicular insertion 0.5–1.0 cun
Hegu (LI 4)	On the dorsum of the hand, between 1st and 2nd metacarpal bones, in the middle of the 2nd metacarpal bone on the radial side	Perpendicular insertion 0.5–1.0 cun. This point is prohibited in pregnancy
Zulinqi (GB 41)	Proximal to the fourth metatarsophalangeal joint, in the depression lateral to the tendon of m. extensor digiti minimi of the foot	Perpendicular insertion 0.3–0.5 cun

### 2.4. Outcome and follow-up

**Figure 2.**
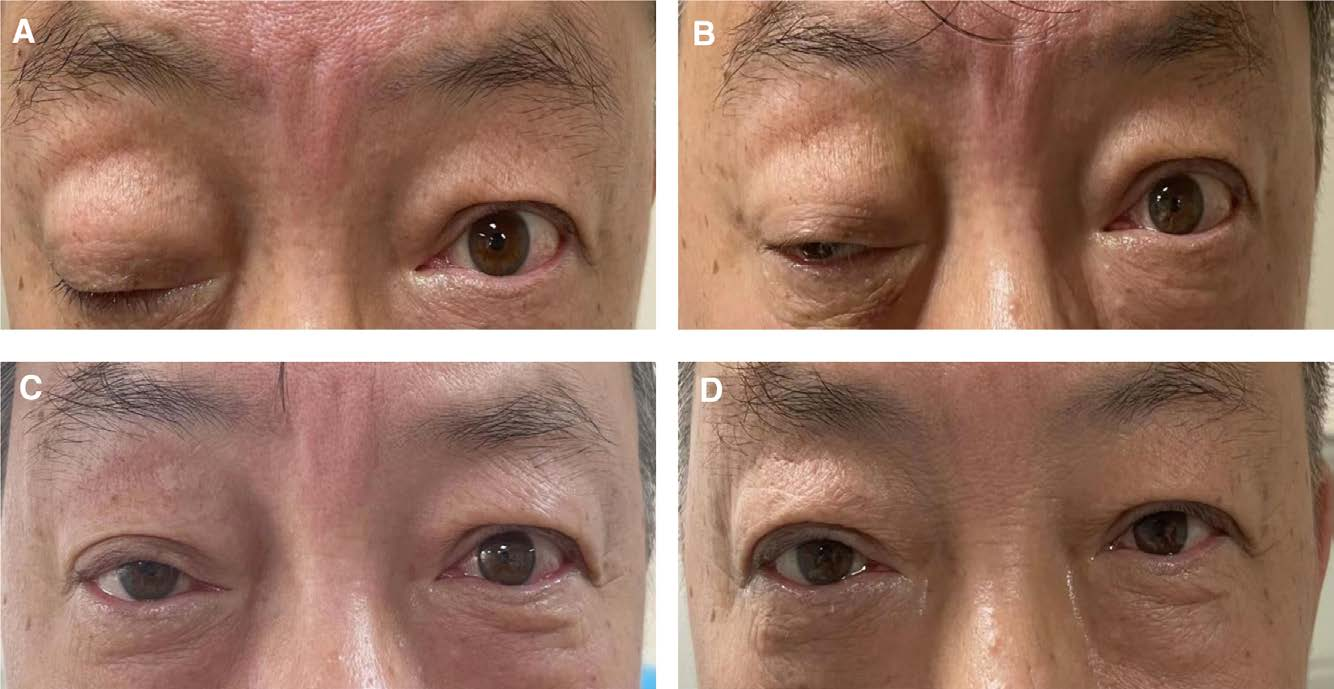
Changes in patient’s eyes (right). (A) Before the treatment. (B) One week after acupuncture treatment. (C) Two weeks after acupuncture treatment. (D) Three weeks after acupuncture treatment.

Prior to acupuncture treatment, the patient exhibited a complete closure of the right upper eyelid and was unable to raise it voluntarily. In addition, the movement in this eye was severely limited, with vertical movement of approximately ±10° and adduction around 10°. The patient also experienced diplopia (Fig. 2A). One week after the acupuncture treatment, the patient could raise the right eyelid by about 3 mm. Furthermore, the right eye adduction was around 30° and vertical movement was approximately ±20°. In addition, a significant improvement in diplopia was observed, with a blood pressure at 124/68 mm Hg (Fig. 2B). Two weeks after acupuncture treatment, the patient’s right eyelid could be raised by around 8 mm, the right eye’s adduction movement was approximately 45°, and the vertical movement around ±30°. The diplopia experienced by the patient was mild, and his blood pressure was measured as 126/72 mm Hg (Fig. 2C). Three weeks after the treatment, the patient almost completely recovered. Namely, the right eyelid could be raised by about 10 mm, right eye adduction was approximately 60° and vertical movement around ±45°. In addition, there was no limitation of movement and no diplopia, and the blood pressure was at 126/74 mm Hg (Fig. 2D). The patient underwent a consistent 3-week acupuncture course and was highly satisfied with the outcomes, not reporting any adverse reactions during the acupuncture treatment period. A telephone follow-up 3 months later revealed that the patient was leading a normal life and there was no recurrence of the symptoms.

After the end of the treatment, the patient reported that: “After receive the first acupuncture treatment, the muscles surrounding the eyes felt relaxed and the patient was able to slightly raise the eyelids. Following the final treatment, the diplopia vanished and the eye was free to move.”

## 3. Discussion

The oculomotor nerve originates from the oculomotor nucleus located in the midbrain tectum and comprises motor and parasympathetic fibers. Motor fibers emerge from the lateral nucleus of the oculomotor nerve, innervating the ipsilateral levator palpebrae superioris, superior rectus, medial rectus, inferior oblique, and inferior rectus muscles, facilitating the elevation of the upper eyelid and ocular motility.^[1,8]^ The parasympathetic fibers arise from the Edinger-Westphal nucleus and the Perlia’s nucleus. They innervate the ipsilateral pupillary sphincter and ciliary muscles and play roles in pupil constriction and the accommodative reflex.^[1,8]^ Long-term hypertension in the patient’s history has accelerated endothelial cell aging and vascular aging, altered vascular hemodynamics, and predisposed the patient to microvascular ischemia.^[9,10]^ Microvascular ischemia leads to the release of high mobility group box 1 by neurons, reactive microglia, and reactive astrocytes.^[11]^ High mobility group box 1 stimulates microglial polarization and the formation of pro-inflammatory M1-type cells, which causes neuroinflammation in ischemic areas.^[12]^ Concurrently, microvascular ischemia reduces superoxide dismutase activity and elevates malondialdehyde activity, triggering cellular oxidative stress responses that lead to neuronal damage.^[13]^ The patient exhibited impairment of motor fibers of the oculomotor nerve due to microvascular ischemia, which is known to lead to inflammation and oxidative stress response in nutrient arteries. Nevertheless, no damage was observed to the parasympathetic fibers. This can be explained by the specific anatomy of the oculomotor nerve, which has central nutrient arteries that supply blood. These arteries do not primarily serve the superficial layer, which is mainly composed of parasymphathetic fibers.^[8]^

Based on the patient’s medical history, physical examination, and prior ancillary tests, we excluded the possibility of such etiologies of the oculomotor nerve palsy as trauma, infection, diabetes, autoimmune diseases, intracranial lesions, and congenital disorders. Reanalyzing possible reasons for the patient’s condition, we found that the oculomotor nerve palsy in the patient primarily involved the motor fibers, manifesting mainly by ptosis of the upper eyelid, limited ocular mobility, and diplopia. However, the parasympathetic fibers remained almost unaffected. Additionally, the patient had a long history of hypertension and cerebral arteriosclerosis. Therefore, we speculated that the patient might be suffering from isolated ischemic oculomotor nerve palsy due to microvascular ischemia.^[1,14]^

Acupuncture treatment originates from the Chinese meridian theory. Acupoints can be applied to treat diseases in their location as well as in the parts where their meridians pass through. Since the foot greater yang bladder channel (BL), foot lesser yang gallbladder channel (GB), foot yang brightness stomach channel (ST), hand yang brightness large intestine channel (LI), and hand lesser yang triple energizer channel (TE) have a close connection with the eyes along the course of a meridian, we chose acupoints around the eyes such as Jingming (BL 1), Cuanzhu (BL 2), Sizhukong (TE 23), and Chengqi (ST 1), coupled with distal limb points Fengchi (GB 20), Waiguan (TE 5), Hegu (LI 4), and Zulinqi (GB 41) for treatment. Recent studies have revealed that acupuncture around the eye acupoints can increase the average blood flow of the ophthalmic artery and promote reperfusion of local capillaries.^[15,16]^ Acupuncture interventions reduce the content of angiotensin II in the blood, enhance the bioavailability of nitric oxide, and promote capillary relaxation, facilitating reperfusion to improve nerve function after injuries caused by microvascular ischemia.^[17]^ Meanwhile, studies have found that acupuncture at Waiguan (TE 5) can activate cortical areas responsible for vision (Brodmann areas BA 18 and 19), improve cerebral cortical blood flow, and promote the recovery of nerve functions in damaged brain areas.^[18]^ Furthermore, some researchers suggest that acupuncture treatment can induce: an increase in the expression of brain-derived neurotrophic factors and glial cell-derived neurotrophic factors, elevated IL-10 levels, and reduced TNF-α and IL-6 levels.^[19]^ These effects inhibit microglial cell polarization, alleviate inflammatory responses, and enhance the secretion of neurotrophic factors. This could represent an intervention pathway through which acupuncture treatment inhibits the polarization of microglial cells into M1 phenotype triggered by microvascular ischemia. However, further research is needed to confirm this thesis.

In our study, we observed that acupuncture treatment facilitated the control and management of the patient’s blood pressure. This effect may be associated with the activation of brain regions, including the paraventricular nucleus of the hypothalamus, arcuate nucleus, periaqueductal gray matter in the ventrolateral funiculus, and rostral ventrolateral medulla after acupuncture.^[20–22]^ These activations reduce the excitability of the sympathetic nervous system and enhance vagal activity. The research has demonstrated that acupuncture at Fengchi (GB 20) can increase the expression of neuronal nitric oxide synthase in the arcuate nucleus and periaqueductal gray matter surrounding the ventrolateral aqueduct, inhibiting the activity of the sympathetic nervous system, thereby reducing blood pressure.^[23]^

## 4. Conclusion and limitation

Our findings suggest that acupuncture therapy can accelerate the recovery rate of the condition, improve the quality of life, manage blood pressure in patients, and do not manifest any significant adverse reactions. Acupuncture treatment may serve as an effective supplementary and alternative therapy for isolated ischemic oculomotor nerve palsy.

However, this case study represents an individual instance, and further confirmation of its clinical efficacy necessitates ongoing, multicenter studies with larger sample sizes. There was no long-term efficacy observation, despite the fact that we followed up with the patient for 3 months in this report.

## Acknowledgements

We are grateful for the patient’s trust and recognition of us. Thank you every member for their hard work.

## Author contributions

**Data curation:** Songhao Ning.

**Writing – original draft:** Guan Xu.

**Writing – review & editing:** Kelin He, Xiao Ye, Quanai Zhang.
